# Effects of partial demand uncertainty reduction on private equity financing in small and medium-sized enterprises: A supply chain perspective

**DOI:** 10.1371/journal.pone.0295713

**Published:** 2024-03-28

**Authors:** Jie Deng, Li Yao, Mengyi Chen, Qingsong He

**Affiliations:** 1 School of Accountancy, Chongqing Technology and Business University, Chongqing, China; 2 School of Business Administration, Chongqing Technology and Business University, Chongqing, China; 3 School of Economics and Management, Harbin Institute of Technology (Weihai), Weihai, China; Harbin Institute of Technology, CHINA

## Abstract

The effect of demand uncertainty reduction (DUR) on supply chain management has received tremendous attention. From a financial perspective, studying the impact of DUR is equally significant. This study explores the relationship between DUR and private equity (PE) financing in retail enterprises within a supply chain, which comprises a dominant supplier and a subordinate retailer. This article establishes decision models for a retailer backed by PE under three market demand conditions: range, mean, and range with mean. The study further investigates the impact of partial demand uncertainty reduction (PDUR) on the retailer and PE through comparative analysis of these scenarios. To address incomplete market demand information during the decision-making process, the study employs the minimax regret criterion to construct and solve the model. An intriguing finding of this study is that contrary to intuition, PDUR not only fails to promote PE but also reduces the retailer’s willingness to finance and decreases the asset size for both the retailer and PE. In addition, the better the growth potential for the retail enterprise, the more severe the negative impact brought about by PDUR. Moreover, the impact of PDUR on supplier and supply chain performance is two-fold. PDUR based on range information has a negative impact on the expected profit of the supplier and the supply chain, while PDUR based on mean information has a positive impact on their expected profit.

## 1. Introduction

In the past few decades, with the continuous development of the global economy and the emergence of innovative technologies, private equity and venture capital (PE/VC) have become an important way for small and medium-sized enterprises (SMEs) to obtain funding. According to the "McKinsey Global Private Markets Review 2023" report released by McKinsey & Company, the global private markets fundraising reached $1.2 trillion in 2022, and as of June 30th, 2022, the assets under management of private markets reached $11.7 trillion. Compared to traditional bank loans or bonds, PE/VC financing is more flexible and innovative, and can support SMEs in multiple areas including funding, management, and market. They play an immensely crucial role in the financial system and have a decisive impact on both national economy and enterprise development [1].

However, the benefits of PE/VC are often accompanied by various uncertainties [2], such as demand uncertainty [3], valuation uncertainty [4], policy uncertainty [5] and corporate environmental uncertainty [6], among others. These uncertainties may have an impact on the decision-making, investment returns and exit strategies of PE/VC investments. Li et al. [7] discovered that uncertainty not only directly restricts corporate investment but also indirectly reduces it by increasing financing constraints. As a result, underinvested corporate investment may decrease and investment efficiency may worsen. This negative effect is especially pronounced in small and medium-sized enterprises.

Among these uncertainties, demand uncertainty often has a more direct impact on a company’s financing and operational decisions, as well as its financial performance. CB Insights, an American technology market research company, conducted a survey analyzing the failures of 101 technology startups, and identified the top 20 reasons for their high-growth companies’ collapse. Among these reasons, the primary cause of failure was found to be "no market demand." The survey found that a staggering 42% of failed startups had experienced this problem. Management becomes fixated on developing new ideas and overlooks the potential harm that can result from a lack of market information. According to Pezza’s [8] research, demand uncertainty places both internal and external pressures on a company’s operations. Internally, more inventory investment is required to buffer against shortages, while externally, uncertain sales revenues make it more difficult for a company to sustain its financial feasibility. Therefore, demand uncertainty has the greatest impact on a company’s financial performance, especially its cash position. Wang et al.’s [9] research further suggests that an increase in demand uncertainty may prompt lenders to charge higher interest rates, which could further impact the decisions and performance of supply chain and lending market participants.

Uncertainty reduction theory suggested that people will use various sources when collecting information to reduce their uncertainty [10]. Gomm [11] noted that reducing uncertainty can reduce investment risk and capital costs, improve financial decision-making, and optimize financing. The effect of demand uncertainty reduction (DUR) on supply chain management has received tremendous attention by researchers. DUR will help firms to reduce the mismatch between supply and demand, thus improving their operational and inventory decisions. Unfortunately, from the financial perspective, the importance of DUR has not received sufficient attention that it deserves [12], especially in terms of how DUR affects equity investment and financing decisions, investment return and exit strategies, and so on, which have not been addressed in the literature.

To illustrate the impact of DUR on equity financing, this study focuses on a typical financial issue in which a capital-constrained retailer seeks access to private equity (PE). This study explores the intersection between operations management and finance, and the analysis is conducted in a supply chain that includes two self-interested members (a supplier and a retailer), engaging in a non-cooperative strategic game. Given that businesses typically spend a great deal of time, effort, and resources to acquire complete information, this often results in a conflict between acquiring information and effectively utilizing time. To address this issue, scholars have explored the concept of partial uncertainty reduction, which involves reducing uncertainty in specific aspects of a situation or problem rather than trying to eliminate all uncertainty. This article primarily examines the effect of partial demand uncertainty reduction (PDUR) on equity financing.

To explore the impact of PDUR on equity financing, this paper constructed three decision models based on the reference to Shi et al.’s [13] method of using range to indicate the level of uncertainty. These models respectively explore the relevant decisions of retailers under situations where the demand range, mean, and both range and mean are known. The situation where both demand mean and range are known can be regarded as a partial reduction in demand uncertainty for the cases where only the demand range or mean is known. By comparing the performance of supplier, retailer, and private equity under these three models, this paper examines:

1. How does the partial reduction of demand uncertainty affect the expected profits of various stakeholders in the context of private equity financing?
2. Does partially reducing the uncertainty of market demand information help to enhance the willingness and efficiency of equity financing?

The remainder of this paper is organized as follows. Section 2 presents a review of the relevant literature. In Section 3, we provided a description of the problem and related assumptions, and presented a benchmark model. In Section 4, we constructed and solved the three main models, and conducted preliminary and brief analysis of the results obtained. In Section 5, numerical studies are conducted to present and compare the effects of PDUR on private equity financing. We conclude the paper in Section 6, and our proofs are provided in the S1 Appendix.

## 2. Literature review

There is currently a wealth of academic research on DUR, and closely related research fields to this article include: the impact of DUR on operations management, the impact of DUR on financial, and research related to partial uncertainty reduction.

Researches on the impact of DUR on operations management usually focuses on the operational decisions and performance of supply chain players, and assumes that these players are free of any capital constraint [12, 14, 15]. For example, Miyaoka and Hausman [16] examined the impact of DUR on expected profits of supply chain members in a decentralized supply chain consisting of a supplier and a manufacturer. They found that when the manufacturer sets the wholesale price, the supplier’s expected profit increases with the reduction of demand uncertainty, while the expected profits of the manufacturer and the supply chain vary with changes in cost parameters. Li [17] studied the impact of DUR on competitive retailers and found that with the reduction of demand uncertainty for a retailer, its own equilibrium profit increases while the equilibrium profit of its competitors decreases. In particular, Begen et al. [18] compared the impacts of demand uncertainty reduction and supply uncertainty reduction on production quantity and cost in the supply chain, and found that relative to exerting supply uncertainty reduction effort, exerting demand uncertainty reduction effort leads to producing more units and lower system cost. Unfortunately, reducing demand uncertainty does not always lead to positive outcomes. According to Li and Petruzzi [19], there are cases where demand uncertainty reduction (DUR) can produce mutually beneficial or unilaterally beneficial outcomes, as well as cases where it can lead to unfavorable outcomes for both parties in a bilateral decentralized supply chain, even if the cost of reducing demand uncertainty is zero.

Another important aspect regarding DUR is how it can influence the decision-making of capital-constrained firms in resolving financial issues. These studies generally assume that capital-constrained supply chains may have multiple sources of financing (primarily debt financing), such as bank credit financing (BCF) and trade credit financing (TCF) [12]. This type of research mainly focuses on two areas. The first area focuses on the impact of DUR on the operational decisions of capital-constrained enterprises [20–22]. For example, under the setting of a price-dependent stochastic market demand, Shi et al. [13] studied how demand uncertainty and capital constraint affect retailers’ integrated ordering and pricing policies towards seasonal products. When faced with relatively low levels of demand uncertainty, a retailer deal with such uncertainty typically sets a lower price than the risk-free retailer. In situations of high demand uncertainty, the retailer will typically order more than the risk-free counterpart. Zhai et al. [23] considered a supply chain consisting of a capital-constrained supplier who can finance with bank credit and a retailer who can place orders before or after the supplier’s production. Their research revealed that the optimal decisions and expected profits of both the supplier and retailer vary based on the degree of demand uncertainty reduction, as well as the differences in exogenously determined wholesale prices.

The second area focuses on the impact of DUR on the effectiveness of BCF and TCF [24–26]. Jing et al. [27] demonstrated the impact of demand uncertainty on the selection of BCF and TCF for a capital-constrained retailer through a set of numerical experiments. When demand uncertainty is low, selecting TCF significantly improves supply chain efficiency, while BCF is more efficient when demand uncertainty is high. Chen et al. [28] also found similar findings that for a retailer with no initial capital, TCF is typically the best option at all levels of demand variability. However, as demand uncertainty increases, the incentives for the manufacturer to offer TCF decrease significantly. Additionally, Deng et al. [29] studied the impact of TCF on competition between retailers in a supply chain with two competing retailers under different levels of demand uncertainty. They found that when both retailers are in financial distress, trade credit exacerbates downstream competition when demand uncertainty is high, while it mitigates competition when demand uncertainty is low.

Balancing the need for decision-making information without being overwhelmed is a difficult task. Additionally, acquiring comprehensive demand information can be both challenging and economically difficult [30]. Langley [31] also suggests that too much information can lead to information overload and analysis paralysis. As a result, scholars have begun to focus on partial uncertainty reduction. In fact, optimizing the allocation of investments to partially reduce parameter uncertainty within a limited budget has always been an important topic in engineering design [32]. In the field of supply chain management, partial uncertainty reduction is usually modeled through a Bayesian approach using observations of demand/supply to reduce the corresponding uncertainty, especially in multi-period newsboy frameworks [33]. For single-period supply chain models, the level of partial uncertainty reduction can also be characterized by the size of the intervals to which uncertain variables belong [13].

This paper is unique compared to existing literature in two main aspects. Firstly, focusing on the impact of partial demand uncertainty reduction on equity financing of capital-constrained firms brings important insights into the decision-making process of such firms. Specifically, decision-making under partial demand information sometimes better aligns with the economic benefit principle of firms. By reducing partial uncertainty, firms are able to make more informed and effective decisions, which in turn can lead to better financial performance and attract more investment. Secondly, prior research on DUR in the field of finance has primarily focused on debt financing. However, as Ferrary and Granovetter [34] noted, uncertainty factors reduce the possibility of obtaining traditional financing resources. Therefore, equity financing often becomes the primary way for firms to raise funds when facing significant uncertainty. It is crucial to understand the impact of partial demand uncertainty reduction on equity financing. By expanding the scope of DUR research to equity financing, we can obtain a more comprehensive understanding of how partial demand uncertainty reduction affects the financing decisions of these firms and how to better support their financial goals. Table 1 presents a comparison of the themes explored in this research with those addressed in related papers.

**Table 1:** Differences between this paper and related literature.

Author	DUR	Partial information	Capital-constrained	Equity financing	Endogenous wholesale price	Minimax regret criteria
[12]	√	×	√	×	√	×
[13]	√	×	√	×	×	×
[16]	√	×	×	×	√	×
[17]	√	×	×	×	×	×
[18]	√	×	×	×	×	×
[19]	√	×	×	×	√	×
[21]	√	×	√	×	√	×
[22]	√	√	√	×	×	√
[23]	√	×	√	×	×	×
[29]	√	×	√	×	√	×
[30]	×	√	×	×	√	×
This study	√	√	√	√	√	√

## 3. Problem description and benchmark model

### 3.1 Problem description and assumptions

Considering a two-echelon supply chain, comprising only a single supplier and retailer, the supplier provides goods to the retailer at a wholesale price *w*, while the supplier itself incurs a unit cost of *c*. The retailer, which operates in an emerging market, is a growing enterprise that faces unpredictable market demand, characterized by *ξ*. Due to the highly uncertain external market environment, the retailer can only obtain limited information about the distribution of market demand. The retailer’s selling price is *p*. Under the supplier-led Stackelberg game, the retailer needs to decide the quantity *q* of goods to be purchased from the supplier, while the supplier needs to decide its optimal wholesale price *w*. This article draws on Shi et al.’s [12] approach, which uses the precision of market demand information to indicate the level of demand uncertainty. For example, at the beginning, the retailer only has information about the mean (range) of demand, and after obtaining further information about the range (mean) of demand, the level of demand uncertainty can be considered reduced.

**Fig 1:**
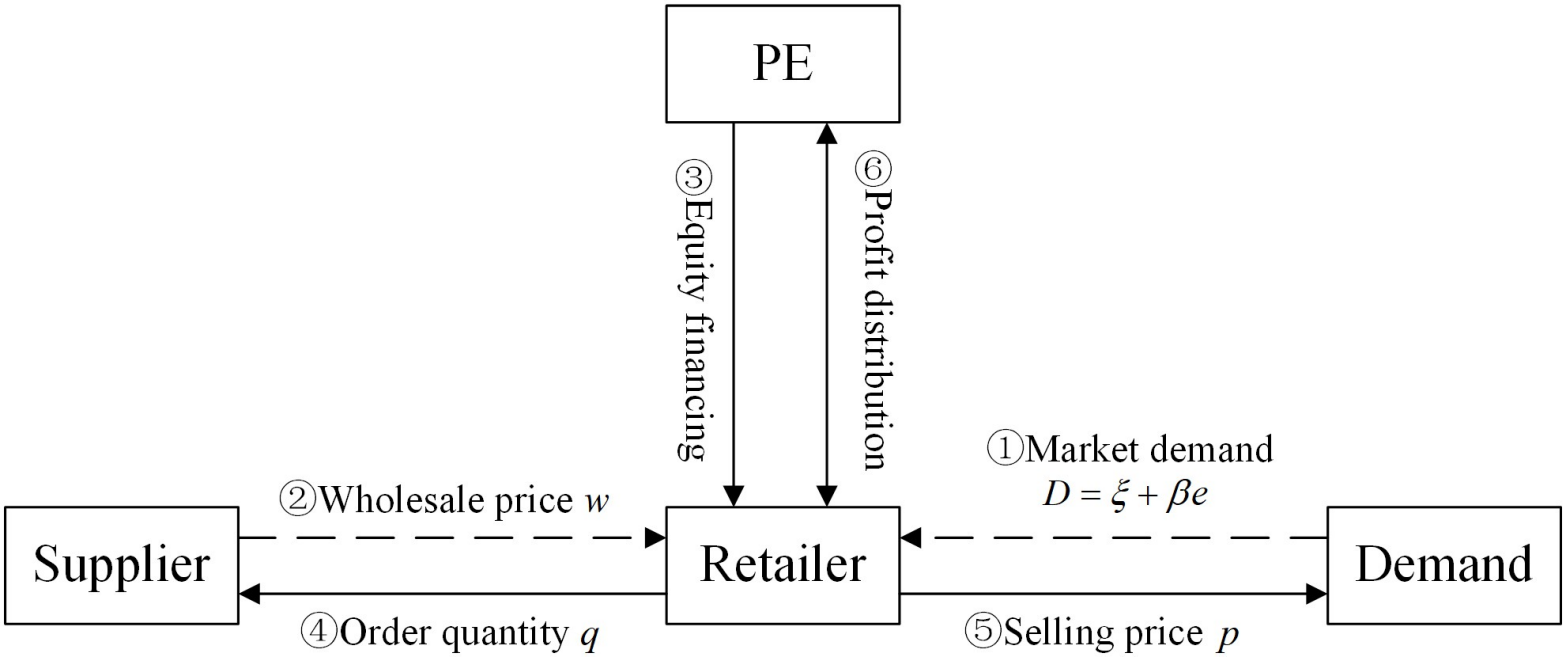
Supply chain operation process.

This article further assumes that the retailer currently faces good market opportunities, and can expand its market and increase market demand through efforts. According to Taylor [35], the new market demand can be represented by *D* = *ξ* + *βe*, where *e* > 0 represents the effort level that the retailer puts into market development, and *β* > 0 represents the demand increment brought by each unit of effort level, which is called the growth factor of the enterprise. Let *V*(*e*) be the effort cost function, which is monotonically increasing with respect to effort level and satisfies the law of increasing marginal cost, and also meets *V*(0) = 0. Without loss of generality, this article assumes *V*(*e*) = 0.5*se*^2^, where *s* > 0 represents the cost coefficient of effort. For growing small and medium-sized enterprises, financial constraints are the main limiting factor for development, often resulting in the company being "conservative and insufficiently enterprising." Assuming that the retailer is unable to bear the cost of market development due to its own financial constraints, in order to seize market opportunities and achieve rapid development, the retailer introduces private equity investment institutions (PE) through equity financing. The above process can be represented by the Fig 1.

Assuming that the PE values the enterprise based on the price-to-book ratio during financing. In fact, among various common valuation methods, the price-to-book ratio valuation method is suitable for supply chain enterprises with a large amount of physical assets and has the advantages of easy access to net asset data that is not easily manipulated. Supposing that after financing, the operational decisions of the retail enterprise are still made by the original shareholders, i.e. the retailers, and their decision-making objective is to maximize their own asset ownership. This article does not take into account the financing costs and taxes, and assumes that the retailer’s expansion of the market will not change the selling price of the product. For the convenience of analysis, other assumptions are added to this article, taking into account the real cases of enterprises such as Gome, JD.com’s deferred payment, Uber, and Touch media, which use all of the financing amount for market development:

1. The product residual value is 0, and the cost of goodwill loss caused by the retailer’s out-of-stock is not considered.
2. The equity financing fund *B* of the retailer is used entirely for market development, that is *B* = 0.5*se*^2^, and the amount of financing determines the extent of the retailer’s market expansion. Other parameter settings of this article are shown in Table 2.

**Table 2:** The list of notations.

Notations	Explanation
*A*	Fixed assets of the retailer before financing
*η*	Retailer’s own funds before financing
*α*	Valuation multiple for the retailer
*θ*_1_	Retailer’s shareholding after equity financing
*θ*_2_	PE’s shareholding after equity financing
*F*	The cumulative distribution function of demand
Γ	The set of all non-negative distribution sets that meet certain conditions

### 3.2 Benchmark model

In the case of incomplete market demand information, decision-makers often adopt a conservative and cautious behavior to develop strategies, which we call robust behavior. Currently, the research methods of this behavior in academia are collectively referred to as robust optimization methods. Among them, the max-min criterion [36] and the minimax regret criterion [37] are two common branches. The former considers profit in the worst-case scenario, which often leads to overly conservative ordering decisions or even non-ordering decisions. According to Perakis and Roels [37], the minimax regret criterion is a less conservative approach. Therefore, this article uses this method to make optimal ordering decisions under incomplete market demand information.

This section considers the development of ordering strategies for retailers without market expansion, i.e., the case where there is no PE participation and *e* = 0 in Fig 1. The unit cost and sales price of the retailer are given by *w* and *p*, respectively. In the minimax regret criterion, "regret" is defined as the difference between the profit that a retailer could have earned with complete information and the profit under incomplete information, which is represented by $\underset{y\geq 0}{\text{max}}\;E_{F}\left[\pi \left(y\right)\right]-E_{F}\left[\pi \left(q\right)\right]$ in formula. *π*(*q*) represents the retailer’s profit. Regret can be seen as the loss caused by incomplete information. Maximum regret is defined as $\rho \left(q\right):=\underset{F\in \Gamma }{\text{max}}\;\left\{\underset{y\geq 0}{\text{max}}\;E_{F}\left[\pi \left(y\right)\right]-E_{F}\left[\pi \left(q\right)\right]\right\}$, which can be understood as the maximum cost a retailer has to pay to obtain accurate demand distribution information. Therefore, the minimax regret criterion is to consider the problem of minimizing the maximized regret value as follows:

$$\rho^{*}=\underset{q\geq 0}{\text{min}}\;\rho \left(q\right)=\underset{q\geq 0}{\text{min}}\;\underset{F\in \Gamma }{\text{max}}\;\left\{\underset{y\geq 0}{\text{max}}\;E_{F}\left[\pi \left(y\right)\right]-E_{F}\left[\pi \left(q\right)\right]\right\}.$$ (1)

Perakis and Roels [37] provided the corresponding results on the minimax regret order quantity under three market demand situations (range, mean, and range with mean). Furthermore, if the supplier and retailer engage in a supplier-dominant Stackelberg game, the supplier should make the optimal wholesale price decision to maximize its profit, which needs to consider the maximization problem: $\underset{w\geq 0}{\text{max}}\;\left(w-c\right)q$. Based on Perakis and Roels [37], we can obtain the following lemma:

**Lemma 1**. If the demand distribution is non-negative, the optimal wholesale price and minimax regret order quantity under three market demand scenarios can be represented in Table 3.

**Table 3:** The optimal decision for the benchmark supply chain under three market demand scenarios.

Demand scenarios	Range	Mean	Range & mean
Optimal decision			
Optimal wholesale price	$\frac{c}{2}+\frac{pr}{2\left(r-l\right)}$	$\frac{p+c}{2}$	$\frac{c}{2}+\frac{p\mu }{2\left(\mu -l\right)}$
Minimax regret order quantity	$\left(1-\frac{c}{p}\right)\frac{r}{2}+\frac{cl}{2p}$	$\left(1-\frac{c}{p}\right)\frac{\mu }{2}$	$\left(1-\frac{c}{p}\right)\frac{\mu }{2}+\frac{cl}{2p}$

## 4. Supply chain operational decisions under different market demand types

According to the previous assumption, a growing retailer can expand its market and increase market demand by making efforts when facing good market opportunities. The new market demand is represented as *D* = *ξ* + *βe*, and the retailer’s order quantity is obviously *q* ≥ *βe*. Due to a lack of funds, the retailer cannot expand the market on their own and therefore introduces a private equity investment institution to increase capital and expand shares, providing all the necessary funds for market expansion. After equity financing, the stake percentage of the retailer in the retail industry can be estimated using the price-to-book ratio valuation method, as $\theta_{1}=\frac{\alpha \left(A+\eta \right)}{\alpha \left(A+\eta \right)+B}$, while the stake percentage of the private equity investor is *θ*_2_ = 1 − *θ*_1_. According to assumption *B* = 0.*5se*^2^, it can be inferred that $e=\sqrt{2B/s}$, and then the net assets of the retailer at the end of the sales period can be represented as:

$$TA_{R}\left(q\right)=\theta_{1}\left(A+\eta +B+p\;\text{min}\;\left\{q,\xi +\beta \sqrt{2B/s}\right\}-wq-B\right).$$ (2)

The net assets of the private equity investor is:

$$TA_{PE}=\theta_{2}\left(A+\eta +B+p\;\text{min}\;\left\{q,\xi +\beta \sqrt{2B/s}\right\}-wq-B\right)-B.$$ (3)

Similar to the benchmark model, we examine the optimal decisions of the retailer and the supplier under three types of market demand. The retailer needs to determine the optimal order quantity to minimize the maximum regret value when demand information is incomplete. In other words, the retailer needs to consider the following minimax regret problem:

$$\rho^{*}=\underset{q\geq 0}{\text{min}}\;\rho \left(q\right)=\underset{q\geq 0}{\text{min}}\;\underset{F\in \Gamma }{\text{max}}\;\left\{\underset{y\geq 0}{\text{max}}\;E_{F}\left[TA_{R}\left(y\right)\right]-E_{F}\left[TA_{R}\left(q\right)\right]\right\}.$$ (4)

The supplier needs to make the optimal wholesale price decision to maximize its profit, with its objective function being:

$$\underset{w}{\text{max}}\;\pi_{s}\left(w\right)=\underset{w}{\text{max}}\;\left(w-c\right)q.$$ (5)

The order of maximization in formula (4) can be rearranged as follows:

$$\begin{array}{l} \rho^{*}=\underset{q\geq 0}{\text{min}}\;\rho \left(q\right)=\underset{q\geq 0}{\text{min}}\;\underset{y\geq 0}{\text{max}}\;\left\{\underset{F\in \Gamma }{\text{max}}\;E_{F}\left[TA_{R}\left(y\right)\right]-E_{F}\left[TA_{R}\left(q\right)\right]\right\}\\ =\underset{q\geq 0}{\text{min}}\;\underset{y\geq 0}{\text{max}}\;\left\{\theta_{1}p\;\underset{F\in \Gamma }{\text{max}}\;\int_{0}^{+\infty }\left(\text{min}\;\left\{y,x+\beta \sqrt{\frac{2B}{s}}\right\}-\text{min}\;\left\{q,x+\beta \sqrt{\frac{2B}{s}}\right\}\right)dF\left(x\right)\right\}+\theta_{1}w\left(q-y\right). \end{array}$$ (6)

Since the retailer has at most the mean of demand, i.e., the first moment information, the maximization problem of the inner layer concerning the distribution function in formula (6) can be redefined as follows:

$$\begin{array}{l} \underset{F\in \Gamma }{\text{max}}\;\int_{0}^{+\infty }\left(\text{min}\;\left\{y,x+\beta \sqrt{2B/s}\right\}-\text{min}\;\left\{q,x+\beta \sqrt{2B/s}\right\}\right)dF\left(x\right)\\ s.t.\;\left\{\begin{array}{l} \int_{0}^{+\infty }dF\left(x\right)=1;\\ \int_{0}^{+\infty }xdF\left(x\right)=\mu ;\\ dF\left(x\right)\geq 0. \end{array}\right. \end{array}$$ (7)

It should be noted that Γ will vary depending on the specific demand information. When only the demand range information is available, Γ represents the non-negative distribution for all ranges between [*l*, *r*]. When only the demand mean information is available, Γ represents the non-negative distribution with a mean of *μ*, and so on.

The optimization problem (7) can be regarded as a linear programming problem with finite constraints and infinite variables. Since the strong duality of linear programming problems always holds, the optimal value of (7) is equivalent to the optimal value of the following dual problem:

$$\begin{array}{l} \underset{\alpha_{0},\alpha_{1}}{\text{min}}\;\alpha_{0}+\mu \alpha_{1}\\ s.t.\;\;\;\alpha_{0}+x\alpha_{1}\geq \text{min}\;\left\{y,x+\beta \sqrt{2B/s}\right\}-\text{min}\;\left\{q,x+\beta \sqrt{2B/s}\right\},\forall x\geq 0. \end{array}$$ (8)

Assuming F* is the distribution that maximizes problem (7), according to strong duality, we obtain the following complementary slackness conditions:

$$\int_{0}^{+\infty }\left[\alpha_{0}+x\alpha_{1}-\left(\text{min}\;\left\{y,x+\beta \sqrt{2B/s}\right\}-\text{min}\;\left\{q,x+\beta \sqrt{2B/s}\right\}\right)\right]dF^{*}\left(x\right)=0.$$ (9)

Therefore, the necessary and sufficient condition for problem (7) to have a non-zero solution (i.e., demand has a non-zero distribution) is:

$$\alpha_{0}+x\alpha_{1}=\text{min}\;\left\{y,x+\beta \sqrt{2B/s}\right\}-\text{min}\;\left\{q,x+\beta \sqrt{2B/s}\right\}.$$ (10)

Let $G\left(y;q\right):=\underset{F\in \Gamma }{\text{max}}\;\left\{E_{F}\left[TA_{R}\left(y\right)\right]-E_{F}\left[TA_{R}\left(q\right)\right]\right\}$. Similar to Perakis and Roels [37], we can obtain the following proposition.

**Proposition 1**. (a) Function *G*(*y*; *q*) is concave on interval *y* ∈ [0, *q*] and interval *y* ∈ [*q*, +∞), but not necessarily globally concave.

(b) Function *ρ*(*q*) is convex, and the optimal order quantity q* = arg min *ρ*(*q*) for the retailer satisfies the following equation:

$$\underset{y\in \left[0,q^{*}\right]}{\text{max}}\;G\left(y;q^{*}\right)=\underset{y\in \left[q^{*},+\infty \right)}{\text{max}}\;G\left(y;q^{*}\right).$$ (11)

**Proof.** See Appendix 1 in S1 Appendix.

Proposition 1 indicates that when seeking the maximum regret value with respect to *y*, it is necessary to consider two cases: *y* ≤ *q* and *y* ≥ *q*. Since function *G*(*y*; *q*) does not have global concavity, the maximum regret value cannot be directly obtained by finding its extreme points. The order quantity that minimizes the maximum regret value should satisfy the following condition: the maximum regret value in the case of insufficient orders is equal to the maximum regret value in the case of excessive orders.

### 4.1 Operational decision-making based on range information

We first assume that the retailer only knows the range [*l*, *r*] of the demand distribution, which can be viewed as "uncertainty budget" [38]. By limiting the uncertain demand to a certain interval, the retailer can to some extent have more precise control over the order quantity. In this case, the equality constraints of problem (7) do not involve the first moment. Through the previous dual process and leveraging the conclusion of Proposition 1, we can obtain the following proposition.

**Proposition 2.** If the demand distribution is non-negative and only the range [*l*, *r*] is known, the optimal order quantity under minimax regret is

$$q^{*}=\beta \sqrt{\frac{2B}{s}}+\frac{w}{p}l+\frac{p-w}{p}r.$$ (12)

**Proof.** Refer to Appendix 2 in S1 Appendix.

From Eq (12), it can be seen that the retailer’s optimal ordering quantity is a monotonically decreasing function of the wholesale price. Further, substituting Eq (12) into the supplier’s profit function yields the supplier’s optimal wholesale pricing decision and the corresponding optimal ordering decision for the retailer.

**Proposition 3.** If the distribution of random demand is non-negative and only the range [*l*, *r*] is known, under the minimax regret decision of the retailer, the optimal wholesale price for the supplier is $w^{*}=\frac{c}{2}+\frac{p\left(r+\beta \sqrt{2B/s}\right)}{2\left(r-l\right)}$, and the corresponding optimal order quantity for the retailer is $q^{*}=\beta \sqrt{\frac{B}{2s}}+\frac{r}{2}-\frac{c\left(r-l\right)}{2p}$.

**Proof.** By substituting Eq (12) into the objective function (5) of the supplier, we can obtain this conclusion.

Comparison Lemma 1 and Proposition 3 indicate that after equity financing, the retailer will increase its ordering level, but correspondingly, the supplier will sell goods at a higher wholesale price. This indicates that in a supplier-dominated supply chain, the overly dominant position of the supplier leads to an imbalance in the bargaining power of both the supplier and the retailer, which may be detrimental to the development of the supply chain. Specifically, if the wholesale price is limited to the level in the benchmark model, it will significantly increase the retailer’s ordering quantity to $q^{*}=\beta \sqrt{\frac{2B}{s}}+\frac{r}{2}-\frac{c\left(r-l\right)}{2p}$.

### 4.2 Operational decision-making based on range and mean information

In this section, we assume that in addition to the range, the retailer can also obtain the mean *μ* of market demand to partially reduce the uncertainty of demand. Then, the ordering quantities *y* and *q* under both complete information and partial information, which appeared in the definition of "regret" in the previous text, should both satisfy $y,q\in \left[l+\beta \sqrt{2B/s},r+\beta \sqrt{2B/s}\right]$. Through the previous duality process, we can obtain the following proposition.

**Proposition 4.** If the demand distribution is non-negative, has a mean of *μ*, and a demand range of [*l*, *r*], then the retailer’s minimax regret order quantity is

$$q^{*}=\left\{\begin{array}{cc} \mu +\beta \sqrt{\frac{2B}{s}}-\frac{w}{p}\left(\mu -l\right), & \frac{1}{2}\leq \frac{w}{p};\\ l+\beta \sqrt{\frac{2B}{s}}+\frac{p\left(\mu -l\right)}{4w}, & \frac{\mu -l}{2\left(r-l\right)}\leq \frac{w}{p}\leq \frac{1}{2};\\ r+\beta \sqrt{\frac{2B}{s}}-\frac{w{\left(r-l\right)}^{2}}{p\left(\mu -l\right)}, & \frac{w}{p}\leq \frac{\mu -l}{2\left(r-l\right)}. \end{array}\right..$$ (13)

**Proof.** See Appendix 3 in S1 Appendix.

Similarly, substituting Eq (13) into the supplier’s profit function yields the following proposition.

**Proposition 5.** If the demand distribution is non-negative, has a mean of *μ*, and a demand range of [*l*, *r*], under the retailer’s minimax regret decision, the supplier’s optimal wholesale price is $w^{*}=\frac{c}{2}+\frac{p}{2\left(\mu -l\right)}\left(\mu +\beta \sqrt{2B/s}\right)$, and the retailer’s optimal order quantity at this wholesale price is $q^{*}=\beta \sqrt{\frac{B}{2s}}+\frac{\mu }{2}-\frac{c\left(\mu -l\right)}{2p}$.

**Proof.** By substituting Eq (13) into the objective function (5) of the supplier, we can obtain this conclusion.

Comparing Propositions 3 and 5, it can be concluded that the partially reduction of demand uncertainty has an impact on the decision-making of supply chain members in the case of the retailer’s equity financing. Consequently, Corollary 1 can be established.

**Corollary 1.** Compared with the case where the retailer only has demand range information, when the retailer obtains additional information about the market demand mean and partially reduces demand uncertainty, the supplier will increase the wholesale price, and the retailer’s order quantity will decrease.

Corollary 1 shows that in a decentralized supply chain, the improvement in information accuracy brought by PDUR enables suppliers to adjust their wholesale prices based on a better understanding of market demand. However, such adjustments may damage the profit and efficiency of the supply chain, and the specific situation needs to be examined through numerical simulation.

Specifically, we consider a particular case: *l* → 0 and *r* → +∞, which is equivalent to the retailer having only the mean information of market demand. In this case, the conclusion of Proposition 3 takes the following form.

**Corollary 2.** If the demand distribution is non-negative with a mean of *μ*, under the retailer’s minimax regret decision, the supplier’s optimal wholesale price is $w^{*}=\frac{p+c}{2}+\frac{p\beta \sqrt{2B/s}}{2\mu }$, and the retailer’s optimal order quantity at this wholesale price is $q^{*}=\beta \sqrt{\frac{B}{2s}}+\left(1-\frac{c}{p}\right)\frac{\mu }{2}$.

Comparing Proposition 3, Proposition 5, and Corollary 2, it can be found that the partially reduction of demand uncertainty always leads to the supplier setting a higher wholesale price. However, it needs to be noted that this does not necessarily mean that the supplier will obtain higher profits. In addition, the impact of the partially reduction of demand uncertainty on the expected profits of supply chain participants and equity financing varies with specific supply chain structures and different interests among participants. Therefore, it is necessary to simulate and explore the impact and changing trends under different conditions through numerical simulation.

## 5. Numerical analysis

In the previous section, we presented the optimal decision-making of a supply chain member under three types of market demand information when a retailer engages in equity financing through a theoretical model. Further, we identified some of the salient features of PDUR in equity financing supply chains by comparing them. In this section, we aim to investigate through numerical simulation: (1) how PDUR impacts equity investment and financing, and (2) how PDUR affects the performance of all participants. Without loss of generality, this paper adopts the method proposed by Andersson et al. [39], and randomly generates a discrete demand distribution within interval [*l*, *r*]. The specific process is as follows:

1. Randomly generate *n* values in the interval [*l*, *r*], and arrange them in ascending order to form sequence *d*_1_, *d*_2_, …, *d*_*n*_;
2. Randomly select *n* points *s*_1_, *s*_2_, …, *s*_*n*_ from the interval [0,1] and normalize them using formula $\rho_{i}=\frac{s_{i}}{\sum_{i=1}^{n}\;s_{i}}$.

By following the above process, we obtain a discrete random distribution with values in *d*_1_, *d*_2_, …, *d*_*n*_ and corresponding probabilities in *ρ*_1_, *ρ*_2_, …, *ρ*_*n*_. Let $\mu =\sum_{i=1}^{n}\;d_{i}\rho_{i}$, and we can obtain the mean of market demand. According to Andersson et al. [39], if *n* is too large, it is easy to obtain a uniform distribution, while a too small value of *n* leads to a too specific distribution. After considering the trade-off, we set *n* = 10 in this paper. After obtaining the random demand distribution, we can substitute it into the optimal solution obtained in the previous section and obtain the performance of each participant under different parameter settings. To avoid extreme cases caused by a single random process, we repeat the above process 10,000 times and take the average as the final reference value for comparison. In the following simulation, we use PE to represent equity investors, R to represent retailers, and S to represent suppliers. All parameter units are set to "1", and the default parameter settings for the simulation are shown in Table 4 unless otherwise specified.

**Table 4:** Default parameter table for simulation.

*A*	*B*	*η*	*p*	*c*	*α*	*β*	*s*	*l*	*r*
2000	2000	1000	50	10	5	10	5	100	2000

From the previous result, the main parameters that affect the optimal decision are the financing amount *B* and the growth factor *β* of the retail company. Therefore, we mainly focus on comparing the results under changes in these two parameters.

**Fig 2:**
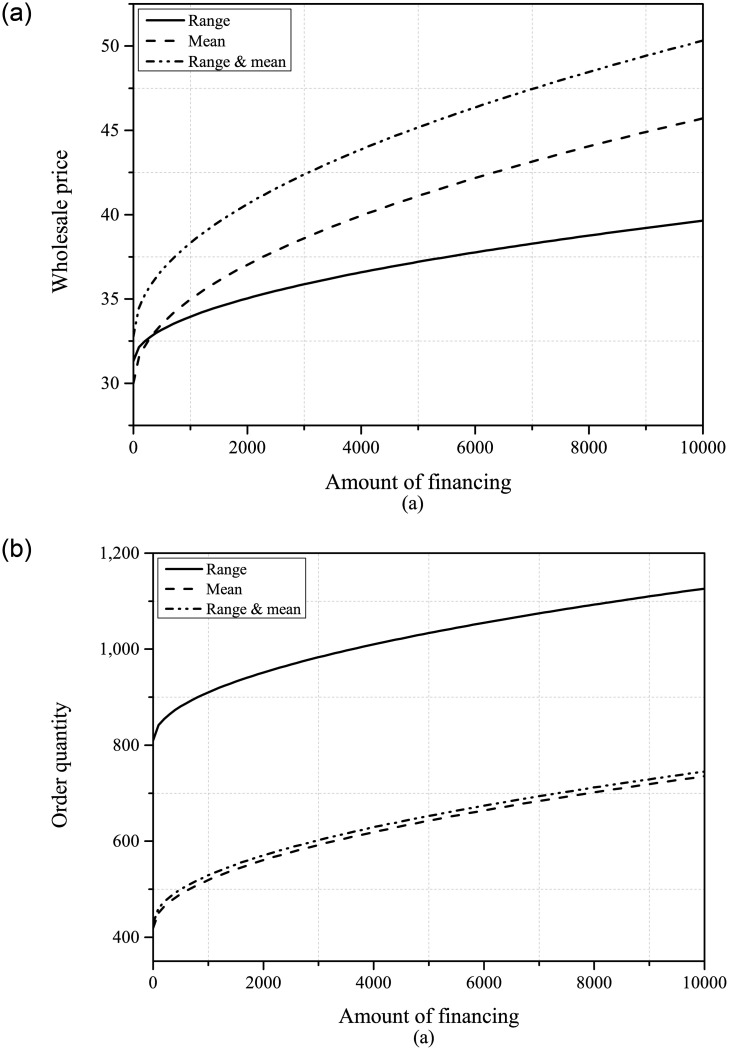
The optimal wholesale price and order quantity (*B* ∈ [100, 10000]).

**Fig 3:**
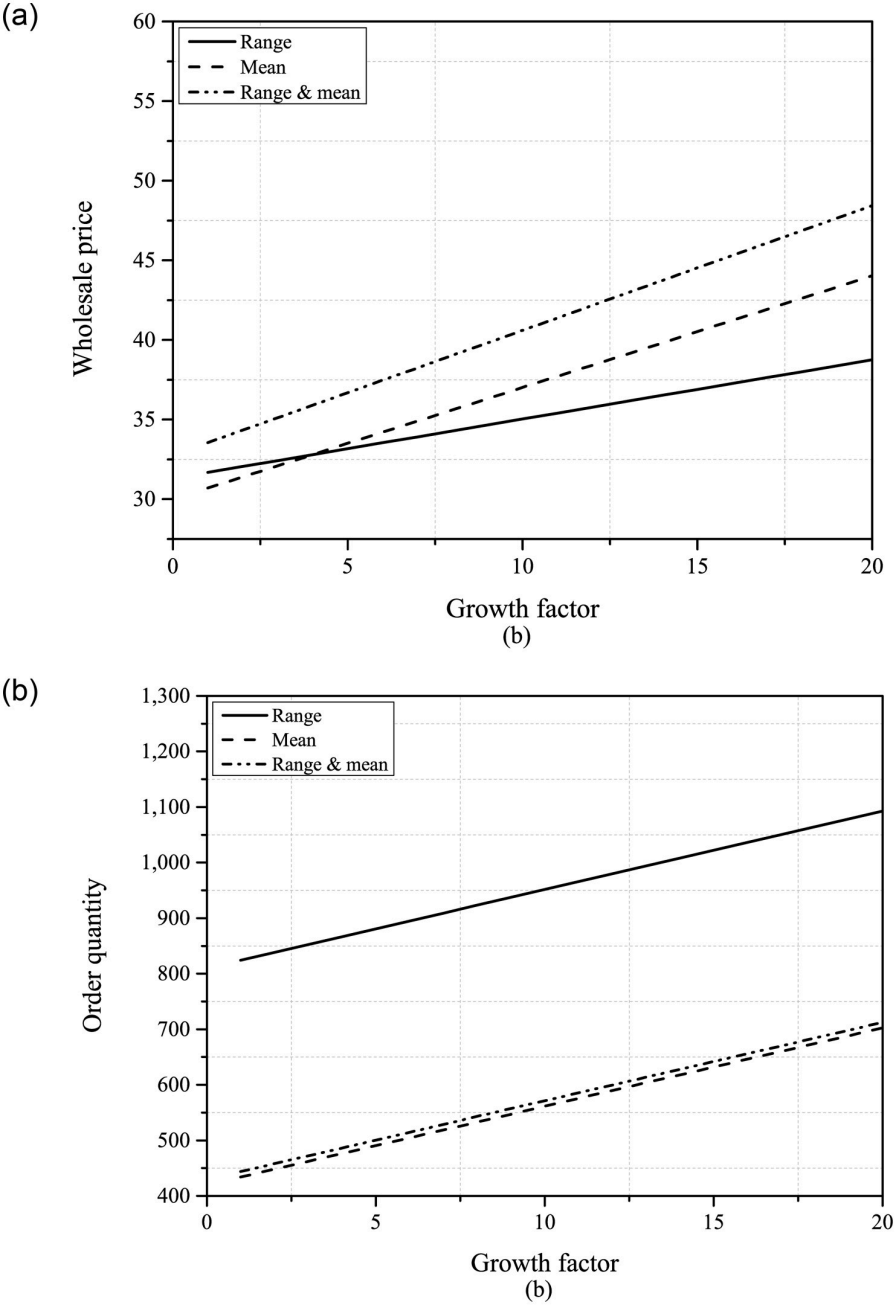
The optimal wholesale price and order quantity (*β* ∈ [1, 20]).

Firstly, we will examine the comparison of the optimal decisions of the supplier and the retailer under the three information scenarios. From Figs 2 and 3, it can be seen that when the supplier has both demand range and mean information, it will set a higher wholesale price. Therefore, PDUR may increase the ordering cost for the retailer and may have a negative impact on its development. Regarding the order quantity, the impact of PDUR is more complicated. If only demand range information is available, PDUR will improve the retailer’s ordering level, but if only mean information is available, PDUR will slightly reduce the retailer’s order quantity.

**Fig 4:**
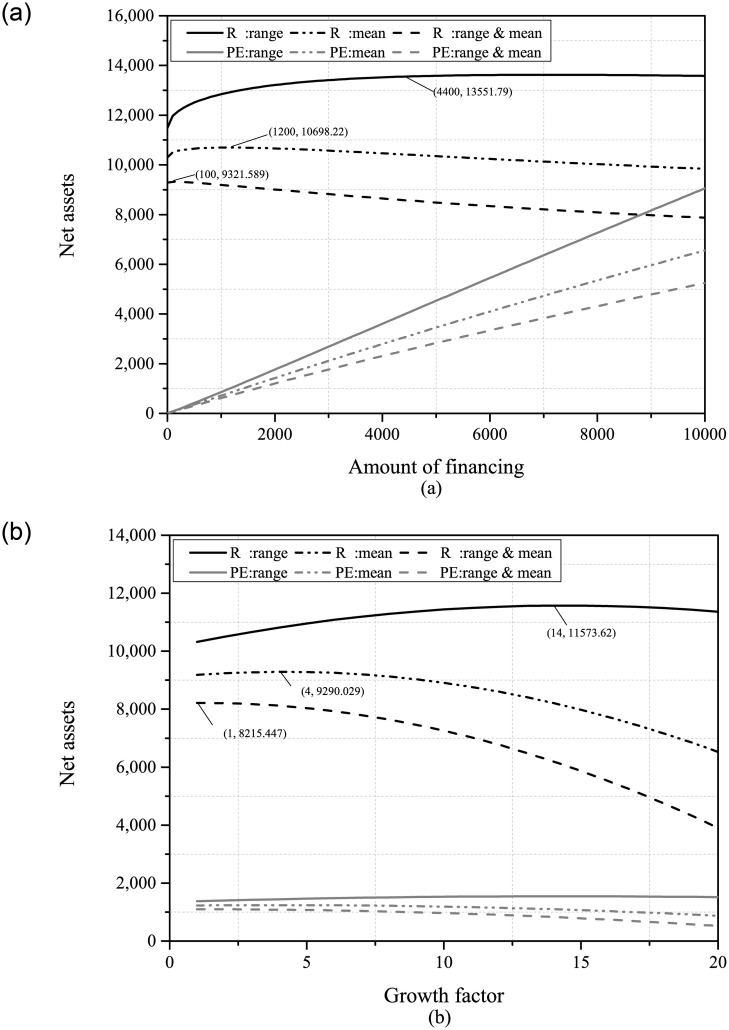
The net assets of the retailer and PE ((a): *B* ∈ [100, 10000]; (b): *β* ∈ [1, 20]).

Secondly, we examined the comparison of net assets between the retailer and the PE under the three information scenarios. Based on the results presented in Fig 4, neither the retailer nor the PE has enough incentive to acquire more market demand information. Regardless of the form of demand uncertainty reduction, PDUR always harms the interests of the retailer and the PE. Furthermore, Panel (b) shows that companies with higher growth potential are more severely affected by the adverse impact brought by PDUR. In particular, Panel (a) indicates that when only demand range or mean information is available, the optimal financing amount of the retailer is higher. However, PDUR will reduce the optimal financing amount of the retailer instead, which means that PDUR lowers the retailer’s willingness to finance. In real-world situations, we can also observe similar occurrences, for instance: Tesla, an American electric vehicle manufacturer, encountered the challenge of uncertain demand in the early stages of the electric vehicle market. As market demand became clearer, Tesla’s willingness to seek financing decreased, and the company began to rely on internal accumulation and debt financing to meet its funding needs. JD.com, a major e-commerce platform in China, also experiences similar situations.

**Fig 5:**
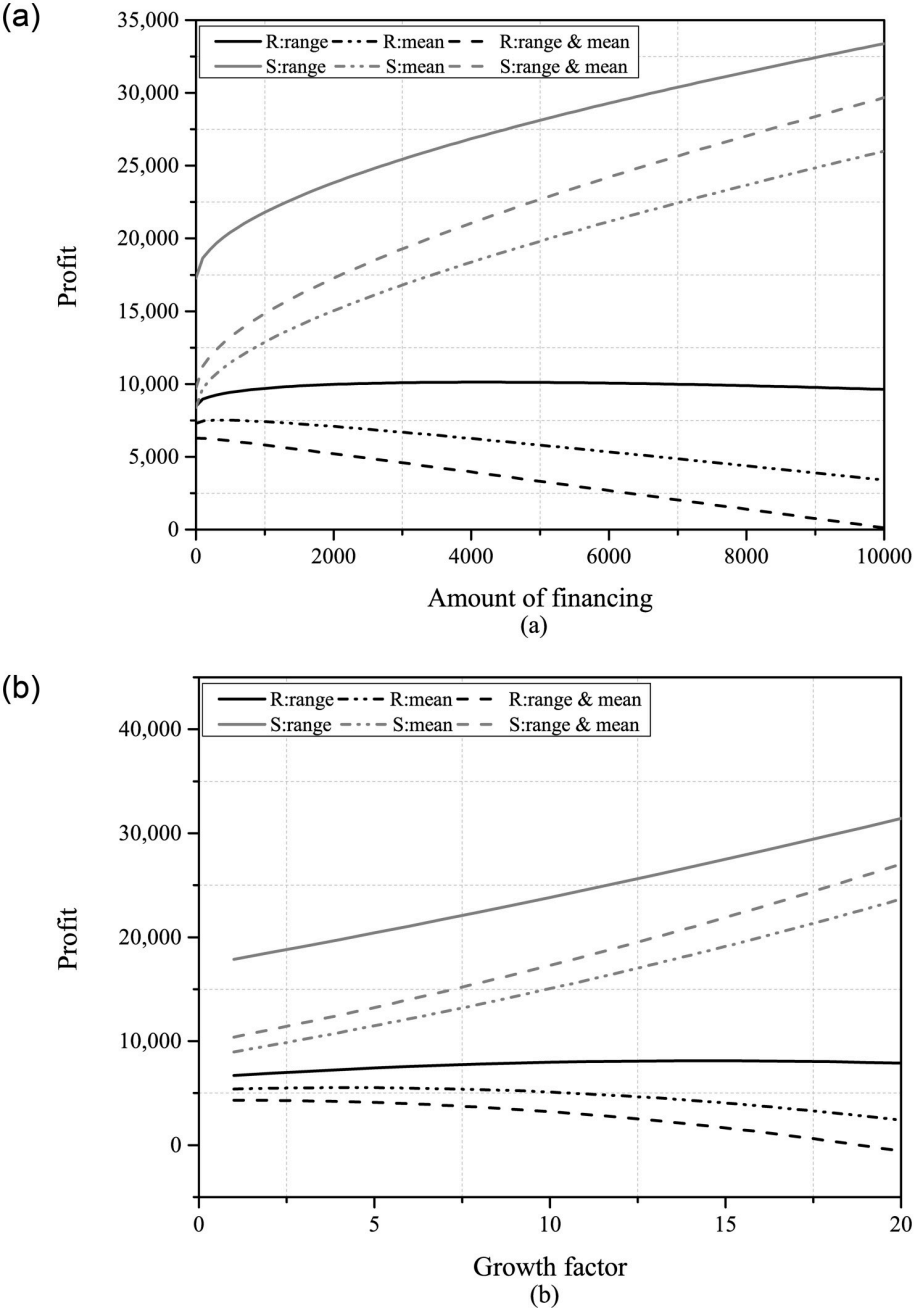
The expected profit of the supplier and the retailer ((a): *B* ∈ [100, 10000]; (b): *β* ∈ [1, 20]).

**Fig 6:**
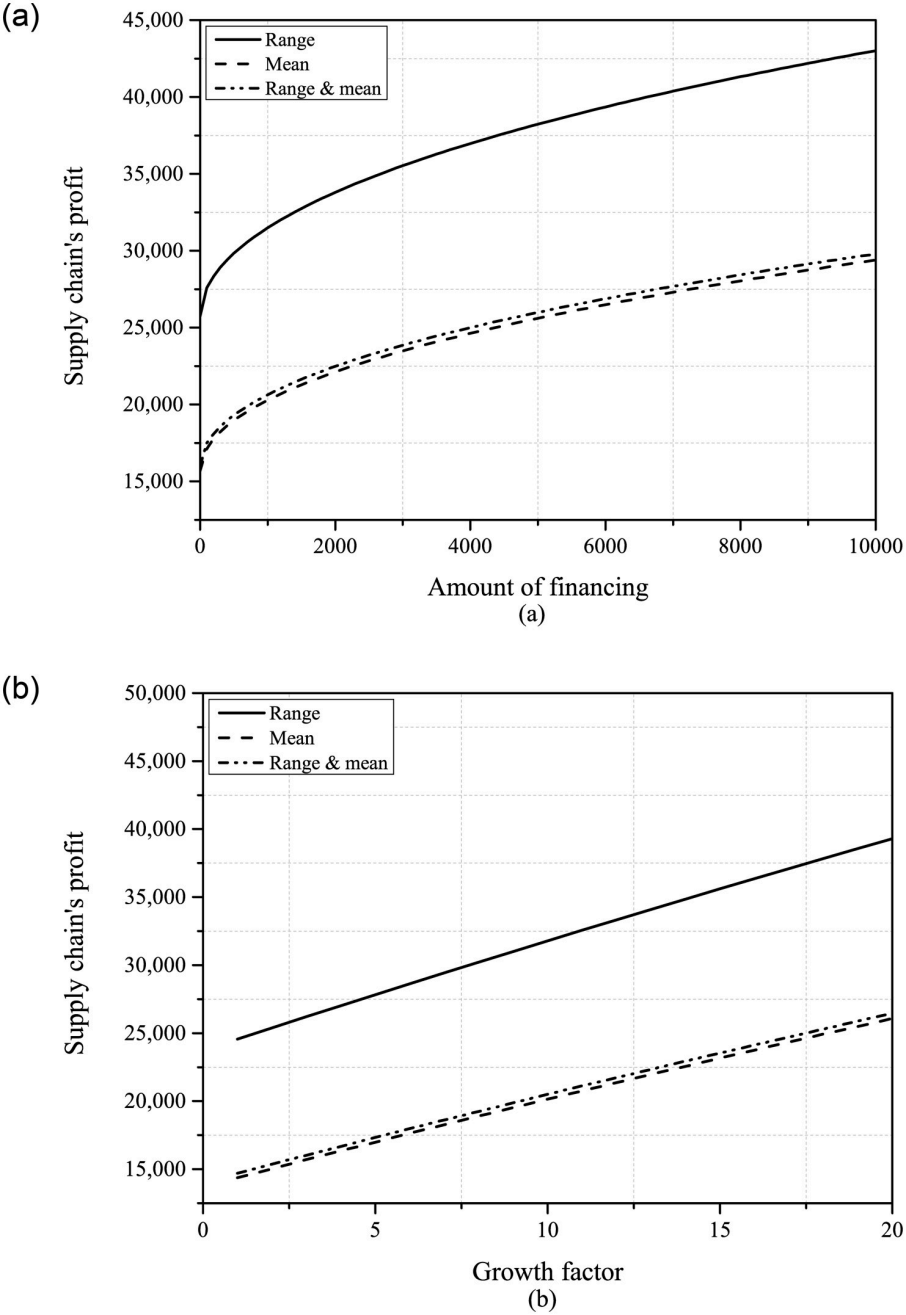
The expected profit of the entire supply chain ((a): *B* ∈ [100, 10000]; (b): *β* ∈ [1, 20]).

Thirdly, we conducted an analysis of how PDUR affects the supply chain and presented the relevant results in Figs 5 and 6. It can be found that, unlike the negative effects that PDUR always brings to the retailer, although reducing uncertainty from only the range information to the situation where both range and mean information are available will harm the interests of the supplier, reducing uncertainty from only the mean to the situation where both mean and range information are available is beneficial to the supplier. From the perspective of the entire supply chain, PDUR greatly reduces the efficiency of the supply chain based on the existing range information. However, in the presence of existing mean information, PDUR will slightly enhance the efficiency of the supply chain. The impact of PDUR on the supply chain shows both positive and negative effects.

Through our simulations, we found that PDUR has different impacts on different participants. Even for the same participant, different forms of PDUR can lead to opposite results. It is worth noting that when obtaining the range and mean information of market demand, the asset size of both the retailer and PE is lower than that of separately obtaining only range or mean information. This phenomenon may be due to the information symmetry and the dominant position of the supplier in the supply chain structure. In this case, the supplier can obtain any market demand information that the retailer obtains, and then, relying on its dominant position in the supply chain, set new wholesale prices that maximize its interests. It is evident that this power structure will lead to a decrease in supply chain efficiency and severely weaken or even alter the value-enhancing role of information in the supply chain. Therefore, in order to achieve higher supply chain efficiency and value for supply chain members and equity investors, the supplier must make appropriate concessions and share benefits.

## 6. Discussion and conclusions

Equity financing is an important way for small and medium-sized enterprises to obtain financial support, but it is vulnerable to the impact of demand uncertainty. This article examines the impact of partial demand uncertainty reduction (PDUR) on equity financing and supply chain participants’ operations in a decentralized supply chain consisting of a single supplier and a capital-constrained retailer. The main findings of the study are as follows:

Firstly, the impact of PDUR on the decisions of a supplier and a retailer is different. Compared to the similar work conducted by Miyaoka and Hausman [16], this work arrived at the same conclusion that in a decentralized supply chain, the expected performance of the retailer always decreases with the reduction of demand uncertainty. However, the difference between this paper and existing literature lies in the fact that the impact of PDUR on the expected profits of the supplier and the supply chain varies depending on the way in which the demand uncertainty reduces. If PDUR is based on demand range information, the expected profits of both the supplier and the supply chain will decrease significantly; if PDUR is based on demand mean information, the expected profits of the supplier and the supply chain will increase.

Secondly, PDUR reduces the willingness of the retailer to finance, which is detrimental to the cooperation and development between the retailer and PE. Different from the study conducted by Shi et al. [12], which focused on the impact of DUR on the choice of debt financing, this paper focuses on the impact of partial DUR on the willingness of enterprises to raise equity financing. The results indicate that when the retailer has demand range or mean information, the optimal equity financing amount for the retailer is higher than the case where they have both range and mean information, and the retailer and PE also have higher net assets in this case. Therefore, PDUR has a negative impact on equity financing for companies facing capital constraints, and the higher the growth potential of the company, the more severe this negative impact will be.

Thirdly, PDUR has a two-sided impact on the overall performance of the supplier and the supply chain. Conducting PDUR based on range information significantly reduces the expected profits of the supplier and the supply chain, while conducting PDUR based on mean information slightly increases the expected profits of the supplier and the supply chain. Overall, when it comes to equity financing for a retailer, it is more advantageous for the overall development of the supply chain to appropriately reduce the dominant position of the supplier in a decentralized supply chain.

Specifically, the above results reflect a phenomenon where the range information of market demand is always the most advantageous information for the retailer and supplier. This provides a reference direction for companies facing uncertain demand markets to conduct market demand information research during equity financing.

Although this article focuses on the impact of PDUR on equity financing and the operation of the supply chain, providing effective references for the retailer’s information selection in obtaining equity financing and rapid growth, it further expands the theoretical research scope of supply chain finance. However, this article did not consider the mutual influence between the effort level of the financed company and equity financing behavior. Moreover, the risks faced by the company during rapid growth are not only demand risks but also high growth risks, mainly manifested as the randomness of the demand increment brought by the efforts made by the company. Blind optimism will occur if the high growth risk of the company is ignored. Therefore, it is very important to take a cautious attitude towards risks and make reasonable decisions when a company conducts equity financing. On the other hand, how to achieve the coordination of the supply chain and improve the efficiency of the supply chain through appropriate supply chain contracts is also a research topic worth paying attention to in PDUR research, and further research is needed to expand it.

## Supporting information

S1 Appendix (DOC)

S1 File Numerical simulation source code. (RAR)
