# Supplementary material for: Effects of partial demand uncertainty reduction on private equity financing in small and medium-sized enterprises: A supply chain perspective
Source: PLoS One. 2024 Mar 28;19(3):e0295713. doi: 10.1371/journal.pone.0295713 (PMC10977682; doi:10.1371/journal.pone.0295713)
Supplement: S1 Appendix — (DOC) [file pone.0295713.s001.doc]

# Appendix

## 1. Proof of Proposition 1

(a) Let$g\left(x,y;q\right):=\min\left\{y,x+\beta\sqrt{2B/s}\right\}-\min\left\{q,x+\beta\sqrt{2B/s}\right\}$, and first consider the convexity and concavity of function$g\left(x,y;q\right)$. It can be proved that function$g\left(x,y;q\right)$is concave on $\left\{\left(x,y\right)|x+\beta\sqrt{\frac{2B}{s}}\geq q,y\geq q\right\}$. In fact, according to the definition of a concave function, $\forall\left(x_{1},y_{1}\right),\left(x_{2},y_{2}\right)\in\left\{\left(x,y\right)|x+\beta\sqrt{\frac{2B}{s}}\geq q,y\geq q\right\}$, and$\forall\lambda_{1},\lambda_{2}\in\left(0,1\right)and\lambda_{1}+\lambda_{2}\text{=}1$, if $\lambda_{1}g\left(x_{1},y_{1};q\right)+\lambda_{2}g\left(x_{2},y_{2};q\right)\leq g\left(\lambda_{1}x_{1}+\lambda_{2}x_{2},\lambda_{1}y_{1}+\lambda_{2}y_{2};q\right)$ is true， then$g\left(x,y;q\right)$is a concave function.

$\begin{matrix} \lambda_{1}g\left(x_{1},y_{1};q\right)+\lambda_{2}g\left(x_{2},y_{2};q\right) \\ \text{=}\min\left\{\lambda_{1}y_{1},\lambda_{1}x_{1}+\lambda_{1}\beta\sqrt{\frac{2B}{s}}\right\}+\min\left\{\lambda_{2}y_{2},\lambda_{2}x_{2}+\lambda_{2}\beta\sqrt{\frac{2B}{s}}\right\}-\left(\lambda_{1}+\lambda_{2}\right)q \\ \leq\min\left\{\lambda_{1}y_{1}+\lambda_{2}y_{2},\lambda_{1}x_{1}+\lambda_{2}x_{2}+\beta\sqrt{\frac{2B}{s}}\right\}-\min\left\{q,\lambda_{1}x_{1}+\lambda_{2}x_{2}+\beta\sqrt{\frac{2B}{s}}\right\} \\ =g\left(\lambda_{1}x_{1}+\lambda_{2}x_{2},\lambda_{1}y_{1}+\lambda_{2}y_{2};q\right). \end{matrix}$. (A1)

Therefore, $g\left(x,y;q\right)$is a concave function on$\left\{\left(x,y\right)|x+\beta\sqrt{\frac{2B}{s}}\geq q,y\geq q\right\}$. Since concavity is preserved under non-negative weighted integration, $\int_{x+\beta\sqrt{2B/s}\geq q} g\left(x,y;q\right)dF\left(x\right)$is a concave function of$y$on the interval$y\geq q$.Moreover, since$g\left(x,y;q\right)=0$holds when$x+\beta\sqrt{2B/s}\leq q$ under the premise of$y\geq q$, $\int_{0}^{+\infty} g\left(x,y;q\right)dF\left(x\right)$ is a concave function on interval$y\geq q$, and then$\theta_{1}\left[p\int_{0}^{+\infty} g\left(x,y;q\right)dF\left(x\right)+w\left(q-y\right)\right]$is a concave function on interval$y\geq q$. Also, since finding the maximum value over a convex set preserves concavity, $G\left(y;q\right)$is a concave function on interval$y\in\left[q,+\infty\right)$. Similarly, it can be proved that$G\left(y;q\right)$is a concave function on interval$y\in\left[0,q\right]$.

(b) Firstly, we prove that $E_{F}\left[TA_{R}\left(q\right)\right]$ is a concave function with regard to$q$. It is easy to prove that the second derivative of$E_{F}\left[TA_{R}\left(q\right)\right]$is less than zero, so $E\left[\pi_{TA}\left(q\right)\right]$is a concave function. Correspondingly,$E_{F}\left[\pi_{TA}\left(y\right)\right]-E_{F}\left[\pi_{TA}\left(q\right)\right]$is a convex function with regard to $q$. By defining$G^{-}\left(q\right)=\max_{y\in\left[0,q\right]} G\left(y;q\right)$and$G^{+}\left(q\right)=\max_{y\in\left[q,+\infty\right)} G\left(y;q\right)$, and using the theorem that maximizing over a convex set preserves concavity, we have$G\left(y;q\right)$, and even$G^{-}\left(q\right)$and$G^{+}\left(q\right)$are convex functions. $\rho\left(y\right)=\max_{y\geq0} G\left(y;q\right)=\max\left\{\max_{y\in\left[0,q\right]} G\left(y;q\right),\max_{y\in\left[q,+\infty\right)} G\left(y;q\right)\right\}$takes the maximum of two convex functions and is also convex. According to the definition, $G^{-}\left(q\right)$is a non-decreasing convex function and$\lim_{q\to0} G^{-}\left(q\right)=0$; $G^{+}\left(q\right)$is a non-increasing convex function and$\lim_{q\to+\infty} G^{+}\left(q\right)=0$, hence $G^{-}\left(q\right)$and$G^{+}\left(q\right)$must have an intersection point, which is the point where$\rho\left(y\right)$ reaches its minimum, and consequently, there is a$G^{-}\left(q^{*}\right)=G^{+}\left(q^{*}\right)$for $q^{*}=\mathrm{argmin}\rho\left(q\right)$. The proposition is proven.

## 2. Proof of Proposition 2

Note that there is$\beta\sqrt{2B/s}\leq y,q$, and let us denote $h\left(x\right)=\min\left\{y,x+\beta\sqrt{2B/s}\right\}-\min\left\{q,x+\beta\sqrt{2B/s}\right\}$. Combining with the feasibility constraint of the dual problem (8), we solve problem (6) in the following two cases.

**Case 1.** $y\geq q$, in which we have

$h\left(x\right)=\left\{\begin{matrix} 0,\text{ }x+\beta\sqrt{2B/s}\leq q, \\ x+\beta\sqrt{2B/s}-q,\text{ }q\leq x+\beta\sqrt{2B/s}\leq y, \\ y-q,\text{ }y\leq x+\beta\sqrt{2B/s}. \end{matrix}\right.$. (A2)

According to the feasibility constraint of the dual problem (8) and equation (10), the probability density that maximizes the original problem must be an unit pulse in the$x\geq y-\beta\sqrt{2B/s}$interval, with the corresponding maximum function value being$y-q$. Therefore, when$y\geq q$, the regret value of the retailer under the worst distribution is:

$G\left(y;q\right)=\theta_{1}\left(p-w\right)\left(y-q\right)$. (A3)

Therefore, $\max_{r+\beta\sqrt{2B/s}\geq y\geq q} G\left(y;q\right)=\theta_{1}\left(p-w\right)\left(r+\beta\sqrt{2B/s}-q\right)$.

**Case 2.** $y\leq q$, in which we have

$h\left(x\right)=\left\{\begin{matrix} 0,\text{ }x+\beta\sqrt{2B/s}\leq y, \\ y-x-\beta\sqrt{2B/s},\text{ }y\leq x+\beta\sqrt{2B/s}\leq q, \\ y-q,\text{ }q\leq x+\beta\sqrt{2B/s}. \end{matrix}\right.$. (A4)

According to the feasibility constraint of the dual problem (8) and equation (10), the probability density that maximizes the original problem is concentrated in the$x\leq y-\beta\sqrt{2B/s}$interval, with its maximum value being0. Therefore, when $y\leq q$, the regret value of the retailer under the worst distribution is:

$G\left(y;q\right)=-\theta_{1}w\left(y-q\right)$. (A5)

Therefore, $\max_{q\geq y\geq l+\beta\sqrt{2B/s}} G\left(y;q\right)=-\theta w\left(l+\beta\sqrt{2B/s}-q\right)$.

According to Proposition 1, the optimal order quantity$q^{*}$is the order quantity that balances the opportunity cost of ordering too little and ordering too much. Therefore, the optimal order quantity satisfies$\theta_{1}\left(p-w\right)\left(r+\beta\sqrt{\frac{2B}{s}}-q^{*}\right)=-\theta_{1}w\left(l+\beta\sqrt{\frac{2B}{s}}-q^{*}\right)$, i.e., $q^{*}=\beta\sqrt{\frac{2B}{s}}+\frac{w}{p}l+\frac{p-w}{p}r$, and the corresponding minimum-maximum regret value is $\rho^{*}=\theta_{1}\frac{w\left(p-w\right)}{p}\left(r-l\right)$. The proof of Proposition 2 is complete.

## 3. Proof of Proposition 4

The order quantities$y$and$q$under complete information and partial information should satisfy $y,q\in\left[l+\beta\sqrt{2B/s},r+\beta\sqrt{2B/s}\right]$. Similarly, we can solve problem (6) in the following two cases.

**Case 1.** $l+\beta\sqrt{2B/s}\leq q\leq y\leq r+\beta\sqrt{2B/s}$, in which we have

$h\left(x\right)=\left\{\begin{matrix} 0,\text{ }l\leq x\leq q-\beta\sqrt{2B/s}, \\ x+\beta\sqrt{2B/s}-q,\text{ }q-\beta\sqrt{2B/s}\leq x\leq y-\beta\sqrt{2B/s}, \\ y-q,\text{ }y-\beta\sqrt{2B/s}\leq x\leq r. \end{matrix}\right.$. (A6)

According to the feasibility constraints of the dual problem (8) and equation (10), the probability density of the maximum distribution of the original problem is concentrated at the intersection point of$\alpha_{0}+x\alpha_{1}$and$h\left(x\right)$, as shown in Fig A1.


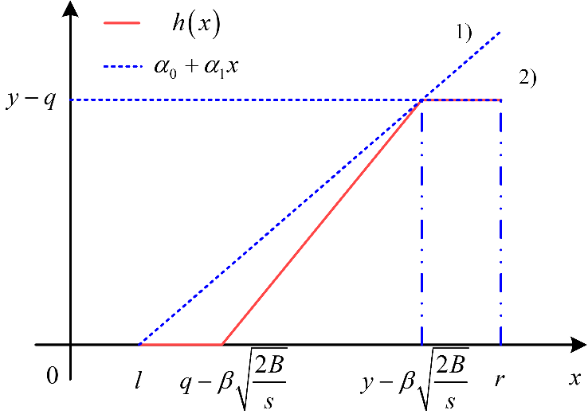


**Fig A1. Possible intersection situations of functions $\alpha_{0}+x\alpha_{1}$and** $h\left(x\right)$

1) When$\alpha_{0}+x\alpha_{1}$and$h\left(x\right)$only have two intersection points, the coordinates of the two intersection points are$\left(l,0\right)$and$\left(y-\beta\sqrt{2B/s},y-q\right)$. Based on the intersection information, $\alpha_{0}=-\frac{\left(y-q\right)l}{y-\beta\sqrt{2B/s}-l}$and$\alpha_{1}=\frac{y-q}{y-\beta\sqrt{2B/s}-l}$can be obtained. Therefore, according to the strong duality principle, the expected profit function of the original problem (7) under the worst distribution at this time is$\alpha_{0}+\mu\alpha_{1}=\frac{\left(\mu-l\right)\left(y-q\right)}{y-\beta\sqrt{2B/s}-l}$. It should be noted that the mean of market demand is $\mu$, and in case 1), the probability distribution of the original problem (7) is a two-point distribution with the maximum value of $y$, so there is a constraint$\mu\leq y-\beta\sqrt{2B/s}$at this time.

2) $\alpha_{0}+x\alpha_{1}$ is a horizontal line, so$\alpha_{1}=0$and$\alpha_{0}=y-q$. According to the strong duality principle, the expected profit function of the original problem (7) under the worst distribution at this time is$\alpha_{0}=y-q$. Similarly, considering that the minimum value of the probability distribution of the original problem (7) in case 2) is $y$, there is also a constraint $\mu\geq y-\beta\sqrt{2B/s}$at this time.

In summary, when$y\geq q$, the retailer's regret under the worst distribution is: .

$G\left(y;q\right)=\left\{\begin{matrix} \theta_{1}\left(p-w\right)\left(y-q\right),\text{ }l\leq y-\beta\sqrt{2B/s}\leq\mu; \\ \theta_{1}\left(\frac{p\left(\mu-l\right)}{y-\beta\sqrt{2B/s}-l}-w\right)\left(y-q\right),\text{ }\mu\leq y-\beta\sqrt{2B/s}\leq r. \end{matrix}\right.$ (A7)

Function$G\left(y;q\right)$is a continuous function.

Now let's consider$\max_{y\geq q} G\left(y;q\right)$. When $l\leq y-\beta\sqrt{2B/s}\leq\mu$, $G\left(y;q\right)$is a monotonically increasing linear function, and its maximum value is obtained at the right boundary$y=\mu+\beta\sqrt{2B/s}$.

When$\mu\leq y-\beta\sqrt{2B/s}\leq r$, it can be easily proved that$G\left(y;q\right)$is a concave function. Let $\frac{dG\left(y;q\right)}{dy}=0$, then $y^{*}=\beta\sqrt{\frac{2B}{s}}+l+\sqrt{\frac{p\left(\mu-l\right)}{w}\left(q-\beta\sqrt{\frac{2B}{s}}-l\right)}$ is the maximum value point, but this point may not be within the interval$\left[\mu+\beta\sqrt{\frac{2B}{s}},r+\beta\sqrt{\frac{2B}{s}}\right]$.

If$\mu+\beta\sqrt{\frac{2B}{s}}\leq\beta\sqrt{\frac{2B}{s}}+l+\sqrt{\frac{p\left(\mu-l\right)}{w}\left(q-\beta\sqrt{\frac{2B}{s}}-l\right)}\leq r+\beta\sqrt{\frac{2B}{s}}$, that is $\beta\sqrt{\frac{2B}{s}}+l+\frac{w\left(\mu-l\right)}{p}\leq q\leq\beta\sqrt{\frac{2B}{s}}+l+\frac{w\left(r-l\right)^{2}}{p\left(\mu-l\right)}$，$y^{*}\in\left[\mu+\beta\sqrt{2B/s},r+\beta\sqrt{2B/s}\right]$, according to the continuity of$G\left(y;q\right)$, the maximum value of$G\left(y;q\right)$when $y\geq q$ is obtained at $y^{*}=\beta\sqrt{\frac{2B}{s}}+l+\sqrt{\frac{p\left(\mu-l\right)}{w}\left(q-\beta\sqrt{\frac{2B}{s}}-l\right)}$, which is $\max_{y\geq q} G\left(y;q\right)=\theta_{1}\left(\sqrt{p\left(\mu-l\right)}-\sqrt{w\left(q-\beta\sqrt{\frac{2B}{s}}-l\right)}\right)^{2}$. If $\beta\sqrt{\frac{2B}{s}}+l+\sqrt{\frac{p\left(\mu-l\right)}{w}\left(q-\beta\sqrt{\frac{2B}{s}}-l\right)}\leq\mu+\beta\sqrt{\frac{2B}{s}}$, that is$q\leq\beta\sqrt{\frac{2B}{s}}+l+\frac{w\left(\mu-l\right)}{p}$, $y^{*}\in\left[l+\beta\sqrt{2B/s},\mu+\beta\sqrt{2B/s}\right]$, the extremum point is on the left side of the concave function. According to the continuity, when$y\geq q$, the maximum value of$G\left(y;q\right)$ is obtained at $y=\mu+\beta\sqrt{2B/s}$, which is$\max_{y\geq q} G\left(y;q\right)=\theta_{1}\left(p-w\right)\left(\mu+\beta\sqrt{\frac{2B}{s}}-q\right)$.

If $r+\beta\sqrt{\frac{2B}{s}}\leq\beta\sqrt{\frac{2B}{s}}+l+\sqrt{\frac{p\left(\mu-l\right)}{w}\left(q-\beta\sqrt{\frac{2B}{s}}-l\right)}$, that is$q\geq\beta\sqrt{\frac{2B}{s}}+l+\frac{w\left(r-l\right)^{2}}{p\left(\mu-l\right)}$，$y^{*}\in\left[r+\beta\sqrt{2B/s},+\infty\right)$, the extremum point is on the right side of the concave function. According to the continuity, when $y\geq q$, the maximum value of $G\left(y;q\right)$ is obtained at the right boundary$y=r+\beta\sqrt{2B/s}$, which is $\max_{y\geq q} G\left(y;q\right)=\theta_{1}\left(\frac{p\left(\mu-l\right)}{r-l}-w\right)\left(r+\beta\sqrt{\frac{2B}{s}}-q\right)$.

In summary, when$y\geq q$, that is, when the order quantity is too small, the maximum regret of the retailer is:

$\rho\left(q\right)=\left\{\begin{matrix} \theta_{1}\left(p-w\right)\left(\mu+\beta\sqrt{\frac{2B}{s}}-q\right),\text{ }q\leq\beta\sqrt{\frac{2B}{s}}+l+\frac{w\left(\mu-l\right)}{p}; \\ \theta_{1}\left(\sqrt{p\left(\mu-l\right)}-\sqrt{w\left(q-\beta\sqrt{\frac{2B}{s}}-l\right)}\right)^{2},\text{ } \\ \text{ }\beta\sqrt{\frac{2B}{s}}+l+\frac{w\left(\mu-l\right)}{p}\leq q\leq\beta\sqrt{\frac{2B}{s}}+l+\frac{w\left(r-l\right)^{2}}{p\left(\mu-l\right)}; \\ \theta_{1}\left(\frac{p\left(\mu-l\right)}{r-l}-w\right)\left(r+\beta\sqrt{\frac{2B}{s}}-q\right),\text{ }\beta\sqrt{\frac{2B}{s}}+l+\frac{w\left(r-l\right)^{2}}{p\left(\mu-l\right)}\leq q. \end{matrix}\right.$ (A8)

**Case 2.** $l+\beta\sqrt{2B/s}\leq y\leq q\leq r+\beta\sqrt{2B/s}$, in which we have

$$h\left(x\right)=\left\{\begin{matrix} 0,\text{ }l\leq x\leq y-\beta\sqrt{2B/s}, \\ y-x-\beta\sqrt{2B/s},\text{ }y-\beta\sqrt{2B/s}\leq x\leq q-\beta\sqrt{2B/s}, \\ y-q,\text{ }q-\beta\sqrt{2B/s}\leq x. \end{matrix}\right.$$

According to the feasibility constraint of the dual problem (8) and equation (10), the probability density of the distribution that maximizes the original problem is concentrated at the common point of $\alpha_{0}+x\alpha_{1}$and$h\left(x\right)$, as shown in Fig A2.


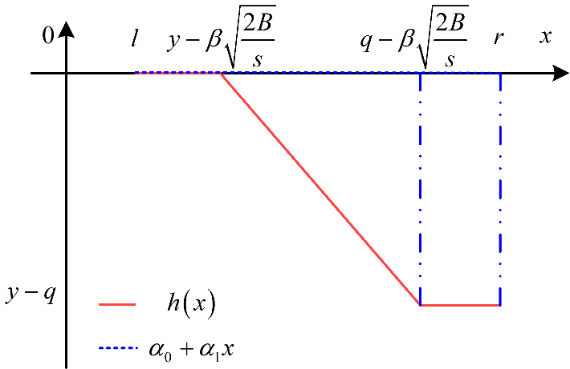


**Fig A2. Possible intersection situations between functions$\alpha_{0}+x\alpha_{1}$and**$h\left(x\right)$

As shown in Figure A2, there is only one possible function$\alpha_{0}+x\alpha_{1}$that satisfies the condition: $\alpha_{0}=\alpha_{1}=0$. Therefore, when$y\leq q$, the maximum regret of the retailer under the worst distribution is$G\left(y;q\right)=-\theta_{1}w\left(y-q\right)$. This is a monotonically decreasing function of$y$, so the maximum value of$G\left(y;q\right)$is obtained at the left endpoint$y=l+\beta\sqrt{2B/s}$. Therefore, when$y\leq q$, that is, when the order quantity is too large, the maximum regret of the retailer is:

$\rho\left(q\right)=\max_{y\leq q} G\left(y;q\right)=\theta_{1}w\left(q-l-\beta\sqrt{2B/s}\right)$. (A9)

According to Proposition 3, the optimal order quantity$q^{*}$is the order quantity that balances the opportunity cost of ordering too little and ordering too much.

When $q\leq\beta\sqrt{\frac{2B}{s}}+l+\frac{w\left(\mu-l\right)}{p}$,

$\rho^{*}=\min_{q\geq0} \max\left\{\theta_{1}w\left(q-l-\beta\sqrt{\frac{2B}{s}}\right),\theta_{1}\left(p-w\right)\left(\mu+\beta\sqrt{\frac{2B}{s}}-q\right)\right\}$. (A10)

According to Proposition 3, the optimal order quantity satisfies $\theta_{1}w\left(q^{*}-l-\beta\sqrt{2B/s}\right)=\theta_{1}\left(p-w\right)\left(\mu+\beta\sqrt{2B/s}-q^{*}\right)$, that is, $q^{*}=\mu+\beta\sqrt{\frac{2B}{s}}-\frac{w}{p}\left(\mu-l\right)$, and the corresponding minimum-maximum regret value is$\rho^{*}=\theta_{1}w\frac{p-w}{p}\left(\mu-l\right)$. The condition $q\leq\beta\sqrt{\frac{2B}{s}}+l+\frac{w\left(\mu-l\right)}{p}$becomes: $\frac{1}{2}\leq\frac{w}{p}$.

When $\beta\sqrt{\frac{2B}{s}}+l+\frac{w\left(\mu-l\right)}{p}\leq q\leq\beta\sqrt{\frac{2B}{s}}+l+\frac{w\left(r-l\right)^{2}}{p\left(\mu-l\right)}$,

$\rho^{*}=\min_{q\geq0} \max\left\{\theta_{1}w\left(q-l-\beta\sqrt{\frac{2B}{s}}\right),\theta_{1}\left(\sqrt{p\left(\mu-l\right)}-\sqrt{w\left(q-\beta\sqrt{\frac{2B}{s}}-l\right)}\right)^{2}\right\}$. (A11)

According to Proposition 3, the optimal order quantity satisfies$\theta_{1}w\left(q^{*}-l-\beta\sqrt{\frac{2B}{s}}\right)=\theta_{1}\left(\sqrt{p\left(\mu-l\right)}-\sqrt{w\left(q^{*}-\beta\sqrt{\frac{2B}{s}}-l\right)}\right)^{2}$, that is, $q^{*}=\beta\sqrt{\frac{2B}{s}}+l+\frac{p\left(\mu-l\right)}{4w}$, and the minimum-maximum regret value is $\rho^{*}=\theta_{1}\frac{p\left(\mu-l\right)}{4}$. The condition$\beta\sqrt{\frac{2B}{s}}+l+\frac{w\left(\mu-l\right)}{p}\leq q\leq\beta\sqrt{\frac{2B}{s}}+l+\frac{w\left(r-l\right)^{2}}{p\left(\mu-l\right)}$becomes:$\frac{\mu-l}{2\left(r-l\right)}\leq\frac{w}{p}\leq\frac{1}{2}$.

When $\beta\sqrt{\frac{2B}{s}}+l+\frac{w\left(r-l\right)^{2}}{p\left(\mu-l\right)}\leq q$,

$\rho^{*}=\min_{q\geq0} \max\left\{\theta_{1}w\left(q-l-\beta\sqrt{\frac{2B}{s}}\right),\theta_{1}\left(\frac{p\left(\mu-l\right)}{r-l}-w\right)\left(r+\beta\sqrt{\frac{2B}{s}}-q\right)\right\}$. (A12)

According to Proposition 3, the optimal order quantity satisfies $\theta_{1}w\left(q^{*}-l-\beta\sqrt{\frac{2B}{s}}\right)=\theta_{1}\left(\frac{p\left(\mu-l\right)}{r-l}-w\right)\left(r+\beta\sqrt{\frac{2B}{s}}-q^{*}\right)$, that is, $q^{*}=r+\beta\sqrt{\frac{2B}{s}}-\frac{w\left(r-l\right)^{2}}{p\left(\mu-l\right)}$, and the minimum-maximum regret value is$\rho^{*}=\theta_{1}w\left(r-l-\frac{w\left(r-l\right)^{2}}{p\left(\mu-l\right)}\right)$. The condition $\beta\sqrt{\frac{2B}{s}}+l+\frac{w\left(r-l\right)^{2}}{p\left(\mu-l\right)}\leq q$becomes: $\frac{w}{p}\leq\frac{\mu-l}{2\left(r-l\right)}$.

In summary, the retailer's optimal ordering decision is:

$q^{*}=\left\{\begin{matrix} \mu+\beta\sqrt{\frac{2B}{s}}-\frac{w}{p}\left(\mu-l\right),\text{ }\frac{1}{2}\leq\frac{w}{p}; \\ \beta\sqrt{\frac{2B}{s}}+l+\frac{p\left(\mu-l\right)}{4w},\text{ }\frac{\mu-l}{2\left(r-l\right)}\leq\frac{w}{p}\leq\frac{1}{2}; \\ r+\beta\sqrt{\frac{2B}{s}}-\frac{w\left(r-l\right)^{2}}{p\left(\mu-l\right)},\text{ }\frac{w}{p}\leq\frac{\mu-l}{2\left(r-l\right)}. \end{matrix}\right.$. (A13)

Proposition 4 is proved.
